# Der p 2.1 Peptide Abrogates House Dust Mites-Induced Asthma Features in Mice and Humanized Mice by Inhibiting DC-Mediated T Cell Polarization

**DOI:** 10.3389/fimmu.2020.565431

**Published:** 2020-11-18

**Authors:** Martin Klein, Luc Colas, Marie-Aude Cheminant, Carole Brosseau, Vincent Sauzeau, Antoine Magnan, Grégory Bouchaud

**Affiliations:** ^1^ UMR INSERM 1087/CNRS 6291, Institut du thorax, Nantes, France; ^2^ School of Medicine, Université of Nantes, Nantes, France; ^3^ UMR INSERM 1064, Centre de Recherche en Transplantation et Immunologie (CRTI), Nantes, France; ^4^ INRAE, Biopolymères Intéractions Assemblages (BIA), Nantes, France; ^5^ Centre Hospitalier Universitaire de Nantes, Service de Pneumologie, Nantes, France

**Keywords:** asthma, immunotherapy, peptide, T lymphocyte (T-cell), dendritic cells, allergy

## Abstract

Asthma is a chronic airway disease often due to sensitization to aeroallergens, especially house dust mite allergens (HDMs). The *Dermatophagoides pteronyssinus* group 2 (Der p 2), is one of the most representative HDM allergens and is recognized by more than 90% of HDM-allergic patients. In mouse models, all asthma-related features can be prevented by prophylactic administration of *Dermatophagoides pteronyssinus* 2-derived peptide (Der p 2.1). However, it is unknown whether it is able to treat well-established asthma in mice and humans. We aimed here to evaluate the efficacy of Der p 2.1 immunotherapy in a mouse, humanized mouse, and asthmatic patients. Asthma related-features were analyzed through airway hyperresponsiveness (AHR), allergen-specific IgE, and lung histology in mice and humanized mice. Immune profile was analyzed using lung and blood from mice and severe asthmatic patients respectively. T cell and dendritic cell (DC) polarization was evaluated using co-culture of bone marrow derived cells (BMDCs) and naïve T cell from naïve mice. Mice and humanized mice both have a reduced AHR, lung tissue alteration, and HDM-specific IgE under Der p 2.1 treatment. Concerning the immune profile, T helper 2 cells (Th2) and T helper 17 cells (Th17) were significantly reduced in both mice and humanized mice lung and in peripheral blood mononuclear cells (PBMCs) from severe asthmatic patients after Der p 2.1 incubation. The downregulation of T cell polarization seems to be linked to an increase of IL-10-secreting DC under Der p 2.1 treatment in both mice and severe asthmatic patients. This study shows that allergen-derived peptide immunotherapy abrogates asthma-related features in mice and humanized mice by reducing Th2 and Th17 cells polarization *via* IL-10-secreting DC. These results suggest that Der p 2.1 peptide immunotherapy could be a promising approach to treat both Th2 and Th17 immunity in asthma.

## Introduction

Asthma prevalence has constantly increased over the last decades, becoming one of the most prevalent airway diseases that affect more than 300 million people worldwide. Despite the efficacy of controller treatments, asthma still results in altered quality of life, morbidity and economic burden (1). Severe asthma represents 5 to 10% of the asthmatic population and results in permanent respiratory limitation, frequent exacerbations and sometimes death (2). Due to the importance of allergy and eosinophilic inflammation in patients displaying a Th2 phenotype (3), biologics such as anti-IgE and anti-IL-5 have been proposed, which are efficacious in 70% of Th2 patients (4–6). However these biologics only block specific immune pathway involved in asthma but do not act on allergy establishment itself, that affects 70% of asthmatic patients (7) and represent the main cause of Th2 asthma with house dust mites (HDM) being the most implied kind of allergens (8). Allergen immunotherapy (AIT) represents the only asthma therapy that can modify the natural course of allergy (9, 10) with a sustained protection, lasting several years after treatment is stopped (11, 12). AIT is based on daily exposure to a high dose of allergen, and according to the European Academy of Allergy and Clinical Immunology (EAACI), to achieve long-term efficacy, it is recommended that a minimum of 3 years of therapy is used depending of ages and type of allergen (13). Induction and activation of regulatory T cells (Tregs) are considered the main mechanism of action of AIT (14). However, the induction of allergen tolerance following AIT requires approximately 1 to 2 years to reach full efficacy; adverse effects due to the use of crude allergen extracts, such as throat irritation, ear pruritus, mouth edema and swollen tongue (12), are frequent. The risk of asthma exacerbations in asthmatic patients still precludes AIT use especially in uncontrolled or partially controlled patients (15).

Based on these observations, AIT efficacy and safety must be improved to make it a full treatment of asthma, including severe or uncontrolled asthma, frequently associated with a non-Th2 difficult-to-treat neutrophilic infiltration. Purified *Dermatophagoides pteronyssinus protein 2* (Der p 2) was shown to be efficient to as a prophylactic AIT to prevent type 2 immunity in murine asthma (16). Genetic engineering led to the development of new therapeutic recombinant hypoallergenic peptides derived from whole allergens displaying interesting properties, such as low IgE induction and T cell reactivity (17). Recombinant hypoallergenic peptides derived from Der p 2 were described to exhibit less *in vivo* allergenicity than Der p 2 whole allergen, while preserving immunogenicity (18, 19). Thus, we previously demonstrated in a mouse model of HDM-induced asthma that the use of Der p 2-derived peptides as a vaccine prevents airway hyperresponsiveness (AHR) and inflammation (20), the two main characteristics of asthma. Recently, non-allergic individuals vaccinated with recombinant hypoallergenic peptide derived from birch pollen allergen Bet v 1 were protected against birch allergy over 2 years with only four subcutaneous injections of the peptide (21). However, the prediction of asthma is still too uncertain to propose AIT as a preventive strategy, and this treatment is proposed mainly once the disease is established. In this paper, we investigated whether Der p 2-derived peptide after HDM sensitization and challenge would decrease HDM-induced asthma features in mice and humanized mice, and the peptide effects was assessed on asthmatic patients’ circulating cells.

## Material and Methods

### Mouse Model

**Figure 1:**
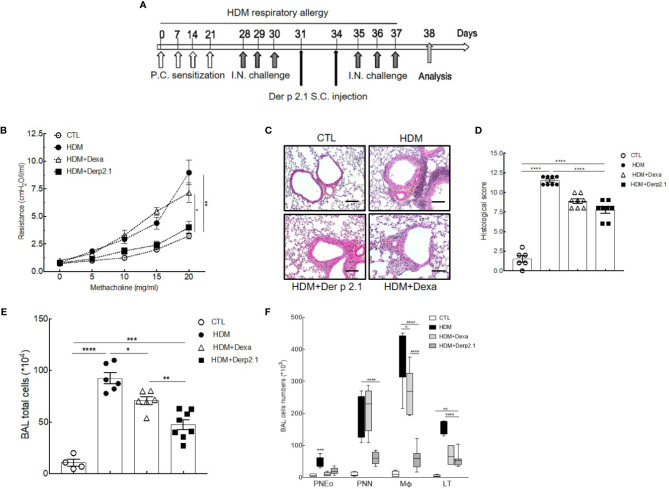
Der p 2.1 decreases asthma features in mouse model of asthma. **(A)** A mouse model of HDM-induced allergic asthma. **(B)** Lung resistances of control (white circles), asthmatic (black circles), and dexamethasone (white triangles) and Der p 2.1-treated (black squares) mice (n = 7–8 mice per group). **(C)** Lungs were stained with hematoxylin eosin coloration: CTL (control), HDM (asthmatic), Dexa (dexamethasone), and Der p 2.1 (Der p 2.1 peptide) (n = 6–8 mice per group). **(D)** Histological slices were scored on 12 points; four points were dedicated to morphologic alteration, and eight points to inflammation (n = 6–8 mice per group). **(E)** and **(F)** BAL lymphocytes, macrophages, eosinophils, and neutrophils (control n = 4, asthmatic n = 6, dexamethasone n = 6, and Der p 2.1 n = 8). Data are presented as the mean ± SEM. *P < 0.05, **P < 0.01, ***P < 0.001 and ****P < 0.0001.

Female BALB/c mice (n = 6–8 mice per group), purchased from Charles River Breeding Laboratories (L’Arbresle, France), were used for all experiments. Mice were housed in a ventilated cage system. The protocol was approved by the Ethics Committee on Animal Experimentation of the Pays de la Loire (accreditation number: 9456). Mice were sensitized on days 0, 7, 14, and 21 by percutaneous application of 500 μg of crude extract of *Dermatophagoides farinae* (Der f, Stallergenes Greer, Antony, France) diluted in 20 μl of dimethyl sulfoxyde (DMSO) (Sigma-Aldrich, Saint Louis, Missouri, USA) on the ears, without any synthetic adjuvant. They were challenged intranasally with 250 μg of *Dermatophagoides farinae* (Der f, Stallergenes Greer, Antony, France) in 40 μl of sterile phosphate buffered saline (PBS) on day 28 to induce AHR and again on days 29, 30, 35, 36, and 37 to enhance AHR. Mice were sacrificed on day 38 (**Figure 1A**).

### Humanized Mouse

Four-week-old NOD scid gamma mouse-IL3-GM-SF (NSG-SGM3) mice (n = 4–6 mice per group) purchased from the Jackson Laboratory’s (Sacramento, California, USA) were irradiated with a dose of 1.5 Gy and anesthetized 6 h later to receive intravenously 0.5 to 1 × 10^5^ cord blood-derived human CD34^+^ hematopoietic stem cells (reference: 2C-101, Lonza, Levallois, France) as previously described (22). After three months, mice were considered humanized when their peripheral blood contained more than 10% of human T cells (**Supplementary Figure 1**). After that, humanized mice were sensitized and challenged with HDM extract as described above. The protocol was approved by the Ethics Committee on Animal Experimentation of the Pays de la Loire (accreditation number: 9456).

### Patients

This study was performed in accordance with recommendations of the Nantes University Hospital Ethical Committee and the Committee for the Protection of Patients from Biologic Risks. All subjects provided written informed consent in accordance with the Declaration of Helsinki. Blood samples were collected from patients included in the EXacerbation PREdictive factors in Severe Asthma (EXPRESA) cohort study (NCT00721097), which is a prospective cohort of severe asthmatic patients sensitized to HDM. Pulmonary function tests, clinical data, and blood samples were collected each month for 1 year. Four severe asthmatic patients were selected within the EXPRESA cohort. Four age- and sex-matched healthy volunteers (HVs), who were free from atopy, asthma, allergic rhinitis, atopic dermatitis, any other inflammatory diseases and treatments were used as controls. All severe asthmatic patients were treated with high dose of inhaled corticosteroids. In HV, % of predicted forced expiration volume (FEV1), and its coefficient of variation was assumed to 100% and age-matched according to reference lung values (23, 24) **(** **Supplementary Figure 2** **)**.

### Treatment

Dexamethasone was diluted in PBS and given intraperitoneally (i.p.) at 1 mg/kg 2 h after each HDM challenge. Derivative peptide (Der p 2.1, amino acids 1-53) was purified as previously described (18). Der p 2.1 was solubilized with a 10 mM NaH_2_PO_4_ (pH 7) solution to a final concentration of 450 μg/ml. Approximately 200 μl of a solution of PBS containing 5 μg of the peptide without adjuvant was injected subcutaneously into the neck of the mice on days 31 and 34 (**Figure 1A**).

### Cell Culture

Bone marrow-derived dendritic cells (BMDCs) were collected from female BALB/c mice shinbones and femurs in a PBS/10% FBS/1% EDTA solution. Cells were counted and cultured for 8 days in non-treated Petri dishes with 10 ml of complete growth medium (RPMI, 1% penicillin/streptomycin, 1% NaPy, 1% HEPES, 10% FBS, 100 μl/L *β*-mercapto ethanol, and 1% L-glutamine) supplemented with 50 μg/ml of purified mouse cytokines GM-CSF (Miltenyi Biotech, Paris, France) and then incubated at 37°C with 5% CO_2_. The cells were harvested on day 8 and cultured for 24 h with 100 μg HDM extract, 10 μg Der p 2.1 or both. Then, 5.10^5^ BMDCs were cocultured for 3 days with 1.10^6^ mouse T CD4^+^ cells sorted from the mouse spleen with an EasySep™ Mouse Naïve CD4+ T Cell Isolation Kit (Stemcell, Grenoble, France) according to the manufacturer’s specifications, and cells were harvested for flow cytometry.

Peripheral blood mononuclear cells (PBMCs) were isolated using a Ficoll gradient. 1.10^6^ PBMCs were incubated in complete growth medium for 3 days for cytokine production and 8 days for T cell polarization with 100 μg HDM alone, 10 μg Der p 2.1 alone and both for a specific re-stimulation, or with phorbol myristate acetate (PMA) 50 ng/ml (Sigma-Aldrich, Saint Quentin Fallavier, France) and Ionomycine 500 ng/ml (Sigma-Aldrich, Saint Quentin Fallavier, France) mix or recombinant Bet v 1 10 μg (Indoor Biotechnologies, Charlotteville, USA) for a non-specific re-stimulation at 37°C with 5%CO_2_. Then, cells were harvested and stained for flow cytometry analysis.

### Immunoglobulin and Cytokine Measurements

Mice blood was collected *via* cardiac puncture 24 h after the last HDM challenge and then centrifuged, and supernatants were frozen at −20°C. The assay for the quantification of HDM-specific IgE was performed in serum samples *via* indirect ELISA. Cytokine concentrations in bronchoalveolar lavage (BAL) supernatants were quantified by Luminex technology (BioPlex 200 system, Bio-Rad Laboratories, Munich, Germany) using a Pro Mouse Group I Cytokine 23-plex kit (Bio-Rad Laboratories, Munich, Germany). Assays were performed according to the manufacturer’s specifications.

### Flow Cytometry

Bronchoalveolar lavage (BAL) was performed with 1 ml of PBS administered intratracheally through a flexible catheter; the lungs were removed and mechanically disrupted to obtain a single-cell suspension and filtered using a 40 μm mesh. BAL and lung cells, BMDCs and human PBMCs were stained with markers described in **Supplementary Methods**.

Data were acquired using DIVA software (BD Biosciences, Paris, France) and analyzed with FlowJo 10.4 (BD Biosciences, Paris, France).

### Airway Hyperresponsiveness

AHR was measured on age-matched (11 weeks) BALB/c female mice using the forced oscillation technique with a FlexiVent (SCIREQ Inc., Montreal, Canada) in response to increasing concentrations of methacholine (0, 5, 10, 15, and 20 mg/ml), as previously described (20). FlexiWare software was used for data analysis.

### Lung Histology

Lungs were fixed in 4% paraformaldehyde for at least 48 h, embedded in paraffin, cut and stained with hematoxylin and eosin for inflammatory scoring. The histological score was calculated blindly based on bronchial morphology and inflammation (12 points) as previously described (25).

### Statistics

Statistical analysis was performed with Prism 7 software (GraphPad Software Inc., La Jolla, USA) using two-way ANOVA followed by Bonferroni correction for repeated measures. Error bars indicate SEM. *P < 0.05, **P < 0.01, ***P < 0.001, ****P < 0.0001.

## Results

### Der p 2.1 Treatment in Mice Model of Asthma

To investigate the therapeutic potential of Der p 2.1 peptide, we measured its effects on AHR, cell inflammation, and histology in the HDM-induced asthma model **(** **Figure 1A** **)**. As expected, asthmatic mice displayed an increase in lung resistance in response to methacholine compared with that of control mice (8.68 *versus* 3.23 cmH_2_O/l/ml, P < 0.001) **(** **Figure 1B** **)**. On the contrary, in mice receiving Der p 2.1, we observed a decrease in lung resistance compared to asthmatic mice (3.63 *versus* 8.63 cmH_2_O/l/ml, P < 0.001) to a control level. Surprisingly, dexamethasone in asthmatic mice did not decrease lung resistance **(** **Figure 1B** **)**. Then, pulmonary lesions were investigated by histology **(** **Figures 1C, D** **)**. Concordant with lung function measurements, asthmatic mice displayed perivascular and peribronchial cell infiltration and epithelial cell hyperplasia **(** **Figures 1C, D** **)**. Der p 2.1 reduced pulmonary lesions (P < 0.0001) in a similar extent as dexamethasone based on lung histological scoring (7.75 *versus* 8.87, P = 0.0918) **(** **Figures 1C, D** **)**. Finally, we explored the effect of Der p 2.1 peptide on lung inflammation **(** **Figures 1E, F** **)**. A dramatic increase in BAL total cells was observed in asthmatic mice (P < 0.0001) compared to controls, which was distributed among lymphocytes (P < 0.0001), macrophages (P < 0.0001), eosinophils (P < 0.0001), and neutrophils (P = 0.0002) **(** **Figures 1E, F** **)**. Mice receiving Der p 2.1 injections displayed a decrease in BAL total cells (P < 0.0001) as well as in all types of inflammatory cells (P < 0.0001 for lymphocytes, P < 0.0001 for macrophages, P = 0.0006 for eosinophils, and P = 0.0016 for neutrophils). By contrast, dexamethasone induced a decrease in BAL total cells (P = 0.0263), lymphocytes (P < 0.0001), and eosinophils (P < 0.0001 and, to a lesser extent, in macrophages (P = 0.0244) but not in neutrophils **(** **Figures 1E, F** **)**. Altogether, our results demonstrate that Der p 2.1 HDM-derived peptide considerably reduced globally the features of asthma, reducing the whole inflammation response, including neutrophilia and thus AHR, two cardinal characteristics shared by all asthma phenotypes. The effect on the neutrophilic component and on AHR was induced by the peptide but not dexamethasone, demonstrating the relevance of the treatment for steroid non-sensitive asthma. Therefore, Der p 2.1 displays highly interesting anti-inflammatory properties that are relevant for severe asthma.

**Figure 2:**
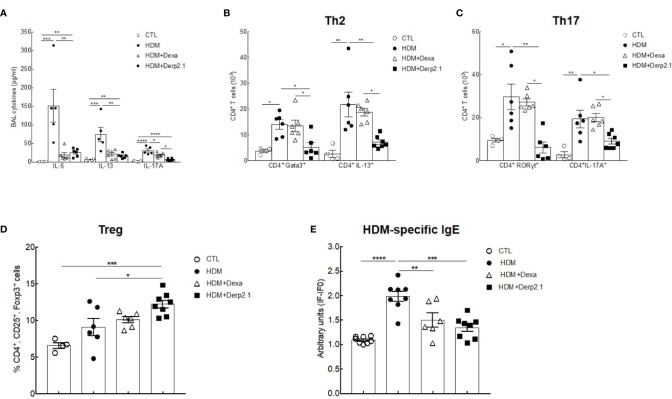
Der P 2.1 treatment decreases cell response in mouse model of asthma. **(A)** BAL cytokines IL-4, IL-5, IL-13, and IL-17 quantification (n = 5*–*6 mice per group). **(B–D)** Lung cells of control (white circles), asthmatic (black circles), and dexamethasone (white triangles) and Der p 2.1-treated (black squares) mice were stimulated for 6 h with HDM extract and stain for Treg, Th2 and Th17 cells (control n = 4, asthmatic, dexamethasone and Der p 2.1 n = 6 mice per group). **(E)** Blood HDM-specific IgE was detected by indirect ELISA. IgE values are expressed in arbitrary units obtained by the ratio of sample fluorescence/basal fluorescence. Data are presented as the mean ± SEM. *P < 0.05, **P < 0.01, ***P < 0.001, and ****P < 0.0001.

To decipher the anti-inflammatory properties of Der p 2.1 peptide, we investigated its effects on the humoral and adaptive responses **(** **Figure 2** **)**. We quantified BAL cytokines and observed significantly decreased concentrations of IL-5 (P = 0.0021 and 0.0012), IL-13 (P = 0.0019 and 0.0043) and IL-17A (P < 0.0001 and P = 0.0334) in Der p 2.1 and dexamethasone-treated mice respectively compared to those in asthmatic mice **(** **Figure 2A** **)**. To assess how T cells were involved in this cytokine modulation, we analyzed pulmonary Treg, Th2 and Th17 cells by flow cytometry by both specific transcription factor expression and cytokine production **(** **Figures 2B, C** **)**. According to assays of BAL, the Der p 2.1 peptide reduced the Th2 response, with a decrease in both CD4^+^GATA3^+^ cells (P = 0.0151) and CD4^+^IL-13^+^ cells (P = 0.049) compared to those in asthmatic mice **(** **Figure 2B** **)**. This treatment also decreased CD4^+^RORyt^+^ and CD4^+^IL-17A^+^ cells, representative of Th17 cells (P = 0.0093 for RORγt and 0.0362 for IL-17A) **(** **Figure 2C** **)**. The levels of both Th2 and Th17 cells in Der p 2.1-treated mice were similar to those in control mice **(** **Figures 2B, C** **)**. However, Der p 2.1 induced an increase in Treg frequency (P = 0.0226) **(** **Figure 2D** **)**. In contrast, dexamethasone had no significant effect on T cells population **(** **Figures 2B–D** **)**. Finally, HDM-specific IgE was measured in the serum of mice as a reflection of the Th2 humoral response **(** **Figure 2E** **)**. As observed with dexamethasone, Der p 2.1 decreased specific IgE levels in asthmatic mice (P = 0.0016) **(** **Figure 2E** **)**. These results show a global effect of Der p 2.1 on the whole inflammatory response by decreasing not only the Th2 response but also the Th17 response and increasing Treg frequency.

### Der p 2.1 Treatment on Mice Cell *In Vitro*

**Figure 3:**
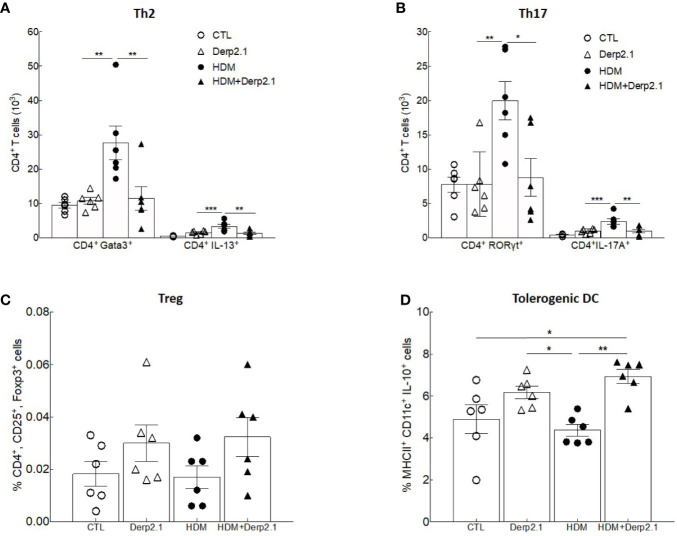
Der p 2.1 treatment inhibits mouse cell polarization *in vitro*. **(A–D)** BMDC and T cells from naïve BALB/c mice polarization in response to no stimulation (white circles), HDM stimulation (black circles), Der p 2.1 stimulation (white squares) and HDM plus Der p 2.1 stimulation (black squares) (n = 6 mice per group). Data are presented as the mean ± SEM. *P < 0.05, **P < 0.01, and ***P < 0.001.

To further elucidate how Der p 2.1 inhibits Th2 and Th17 responses in asthma, we analyzed *in vitro* its effect on T cell differentiation **(** **Figure 3** **)**. We cocultured BMDC and T cells from naïve BALB/c mice in the presence of HDM extract and/or Der p 2.1. When DCs were incubated in the presence of HDM extract, the number of Th2 CD4^+^GATA3^+^ T cells and CD4^+^ IL-13-producing cells increased (P = 0.0026 and P = 0.0002 respectively) compared with those of T cells cultured with control DCs only **(** **Figure 3A** **)**. In contrast, no increase in Th2 cells was observed when DCs were incubated with both HDM extract and Der p 2.1 peptide **(** **Figure 3A** **)**. Similar results were observed in Th17 cells: CD4^+^RORyt^+^ and CD4^+^IL-17A^+^ cells increased (P = 0.0061 and 0.0001 respectively) in the presence of DCs loaded with HDM preparation compared to those in control conditions, whereas this increase was completely abrogated in the presence of Der p 2.1 (P = 0.0128 for RORyt and 0.0050 for IL-17A) **(** **Figure 3B** **)**. As a control, DCs incubated with Der p 2.1 alone did not induce T cell differentiation into Th2 or Th17 cells **(** **Figures 3A, B** **)**. Treg frequency did not vary when DCs were loaded with HDM extract or Der p 2.1 or both **(** **Figure 3C** **)**. These results suggest that Der p 2.1 is able to counteract the HDM ability to induce Th2 and Th17 differentiation of T cells by acting on DC activation. Indeed, in the presence of Der p 2.1 or both HDM extract and Der p 2.1, an increased frequency of CD11c^+^ IL-10^+^ cells was observed (P = 0.0193 and 0.0026 respectively) **(** **Figure 3D** **)**. By contrast, IL-10 production from DCs loaded with HDM extract alone did not increase **(** **Figure 3D** **)**. In conclusion, these results suggest that Der p 2.1 induces IL-10-producing antigen-presenting cells, which could in turn inhibit HDM-induced T cell differentiation.

### Der p 2.1 Treatment on Human Cell *In Vitro*

**Figure 4:**
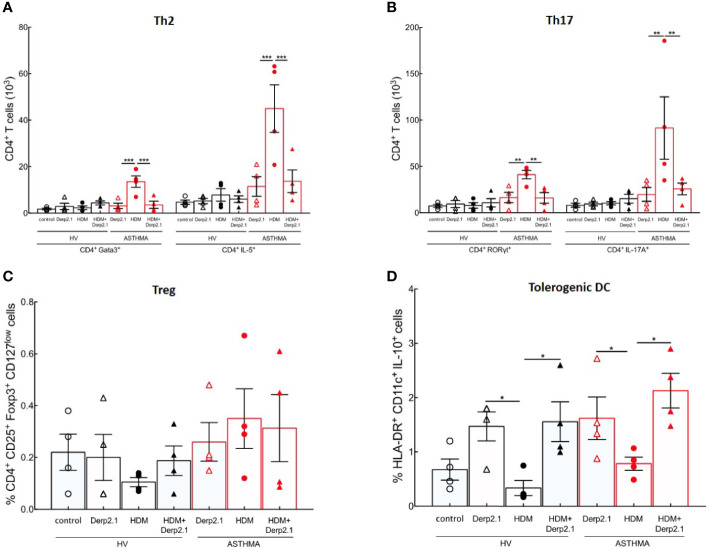
Der p 2.1 inhibits mouse and severe asthma patients’ T cell polarization. **(A, B)** PBMC polarization and cytokine production by HDM−, Der p 2.1− or HDM + Der p 2.1-restimulated from human HV and ASTHMA PBMC, (four patients per group). Data are shown as mean ± SEM. *P <.05, **P <.01, ***P <.001.

Following our previous results on mouse BMDC and T cell polarization, we investigated Der p 2.1 peptide properties on both HV and asthmatic human PBMCs. Peripheral blood mononuclear cells (PBMCs) from healthy volunteers (HVs) and severe asthmatic patients (ASTHMA) were incubated in the presence of HDM, Derp 2.1, or both. As observed in mouse, HDM-re-stimulated PBMCs from asthmatic patients exhibit a strong Th2 and Th17 polarization (P < 0.0001 for GATA3 and P = 0.0003 for RORγt) and cytokine production (P = 0.0001 for IL-5 and P = 0.0040 for IL-17A) without any effect on Treg and IL-10 DC frequency compared to HV (**Figures 4A–D**). Strikingly, Der p 2.1- and HDM/Der p 2.1-re-stimulated PBMCs from HV and asthmatic patients do not polarize nor produce Th2 and Th17 cytokines (**Figures 4A, B** **)**. Treg cell frequency doesn’t change, but we observe an increase of IL-10 DC in both conditions (P = 0.0306 for Der p 2.1 alone and P = 0.0286 for Der p 2.1 with HDM) (**Figures 4C, D** **)**. Non-specific stimulation of both asthmatic and HV PBMCs with a non-relevant allergen (Bet v 1) or with PMA/Ionomycin does not modulate T cell polarization (**Supplementary Figure 3**).

### Der p 2.1 Treatment in Humanized Model of Asthma

**Figure 5:**
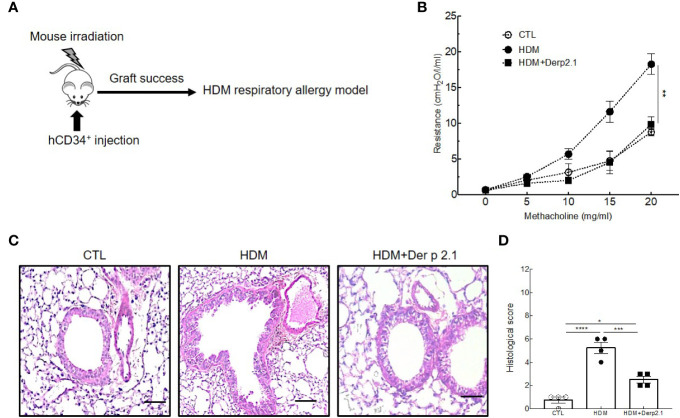
Der p 2.1 decreases asthma features in humanized mice model of asthma. **(A)** A humanized mouse model of HDM-induced asthma. **(B)** Lung resistances of control (white circles), asthmatic (black circles) and Der p 2.1 treated (black squares) mice (n = 4 mice per group). **(C)** Lungs were stained with hematoxylin eosin staining: CTL (control), HDM (asthmatic) and Der p 2.1 (Der p 2.1 peptide) (n = 4 mice per group). **(D)** Histological slices were scored on 12 points; four points were dedicated to morphologic alteration, and eight points to inflammation (n = 4 mice per group). Data are presented as the mean ± SEM. *P < 0.05, **P < 0.01, ***P < 0.001 and ****P < 0.0001.

To confirm our results in a human immune environment and therefore the potential of using Der p 2.1 in a therapeutic setting in humans, we reproduced our experiments using a humanized mouse model of allergic asthma **(** **Figure 5A** **)**. Irradiated mice reconstituted with human CD34^+^ cells and treated with Der p 2.1 peptide displayed a dramatic decrease in airway resistance in response to methacholine compared to that in HDM-exposed mice (9.9 *vs* 18.2 cmH_2_O/l/ml; P = 0.0186) **(** **Figure 5B** **)**. In Der p 2.1-treated humanized mice, the airway resistance in response to methacholine was indeed comparable to control non-asthmatic mice **(** **Figure 5B** **)**. These results were confirmed by anatomical analyses of the lungs **(** **Figures 5C, D** **)**. Likewise, our previous observations showed that humanized mice treated with Der p 2.1 peptide displayed reduced cell infiltrate and epithelial thickening **(** **Figure 5C** **)**. The results blindly quantified indicated that the histological score of Der p 2.1-treated humanized mice was lower than that of asthmatic humanized mice (P = 0.0005) **(** **Figure 5D** **)**. Collectively, our analyses demonstrate that Der p 2.1 peptide efficiently reduces asthma features *in vivo* in asthmatic humanized mice.

**Figure 6:**
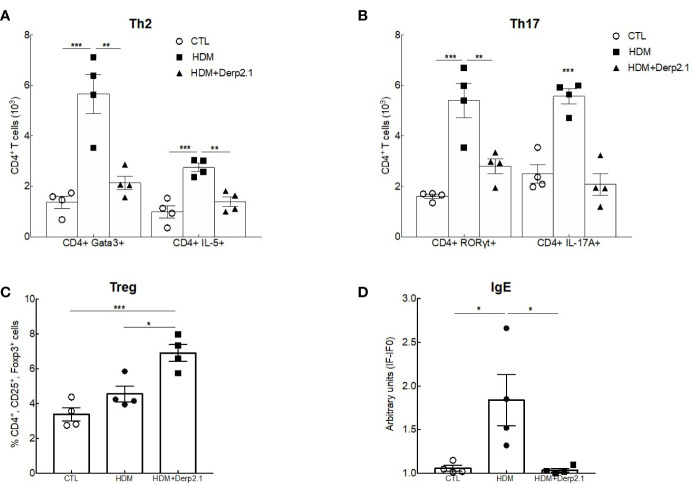
Der p 2.1 modulates cell responses in humanized mice. **(A–C)** Lung Treg, Th2 and Th17 cells were assessed by flow cytometry (n = 4 mice per group). **(D)** Serum HDM-specific IgE was detected by indirect ELISA. IgE values are expressed in arbitrary units obtained by the ratio of sample fluorescence/basal fluorescence. Data are presented as the mean ± SEM. *P < 0.05, **P < 0.01 and ***P < 0.001.

Having shown an effect of Der p 2.1 peptide on lung function and inflammation using humanized mice, we then explored the human immune response in this model **(** **Figure 6** **)**. As expected, both Th2 (CD4^+^GATA3^+^ and CD4^+^ IL-5-producing T cells) and Th17 (CD4^+^ RORγt^+^ and CD4^+^ IL-17A-producing T cells) cell numbers were decreased in the lungs of asthmatic mice treated with Der p 2.1 (P = 0.0020 for GATA3 and 0.0035 for IL-5, P = 0.0061 for RORγt and 0.0002 for IL-17A) compared with those in the lungs of untreated asthmatic mice. Moreover, Der p 2.1-treated mice displayed Th2 and Th17 cell numbers comparable to those of control mice **(** **Figures 6A, B** **)**. The frequency of lung CD4^+^CD25^+^Foxp3^+^ Tregs was not different between control and asthmatic humanized mice, although Treg frequency was increased in the lungs after Der p 2.1 treatment (P = 0.0121) **(** **Figure 6C** **)**. Finally, we measured HDM-specific IgE and observed a decrease in treated mice compared with that in asthmatic mice (P = 0.0281), with a level comparable to that of the control **(** **Figure 6D** **)**. Taken together, these results demonstrate a decrease in both Th2 and Th17 cells in response to Der p 2.1 and an increase in Der p 2.1-induced Treg cells in humanized asthmatic mice.

## Discussion

In the present study, we first confirmed that our mouse model of HDM-induced asthma not only exhibited the allergic asthma features of eosinophilia, Th2 response, and allergen-specific IgE production (26) but also key features of severe asthma, such as neutrophilia, Th17 response, and low steroid sensitivity (27).

In our model, steroid resistance was illustrated by the persistent AHR in asthmatic mice after dexamethasone treatment. As dexamethasone reduced eosinophilia and Th2 cytokines but could not diminish neutrophilia and IL-17A secretion, it is possible that steroid resistance of AHR is related to the IL-17-induced neutrophilic and Th17 contingents (28). Indeed, IL-17A is well known to be associated with elevated levels of neutrophils in the airways and can directly activate smooth muscle cell contraction (29). By contrast, Der p 2 allergen-derived peptide immunotherapy abrogated both Th2- and Th17-related asthma features and established AHR. In addition, lung Treg cell frequency increased under Der p 2.1 treatment. As Treg cells are key players in inflammation resolution, they are candidates to be responsible for the Th2/Th17 modulation induced by the peptide. Accordingly, their increase is associated with success of AIT (30, 31). Moreover, Der p 2.1 seems to act on lung tissue alteration. It is well known that bronchial remodeling is a severe asthma feature (32). Interestingly, our peptide reduced lung infiltrate and decreased epithelium/smooth muscle thickening (**Supplementary Figure 4A**) and decrease alarmin production such as IL-33, thymic stromal lymphopoietin (TSLP) and IL-25 **(** **Supplementary Figures 4B–D** **)**. Based on these observations, we can expect that Der p 2.1 may also decreases smooth muscle cell hyperplasia; however, further investigation like alpha smooth muscle actin staining needs to be realized to measure impact of Der p 2.1 treatment on smooth muscle.

Preclinical assessments of new therapies require animal models reflecting human pathogenesis, but existing differences in the immune physiology of mice and humans limit their interpretation. Previous NSG mouse strains were not relevant enough and failed to fully mimic the human immune system (33, 34). A new model of humanized mice called NSG-SGM3 was able to express functional human Treg, T and B cells, myeloid progenitor, and dendritic cells and mast cells (22, 35–38). NSG-SGM3 HDM-induced asthma model is known to exhibit elevated AHR, a bronchial infiltrate and alteration associated with Th2 inflammation (39). For the first time, we developed a model of HDM-induced allergic severe asthma in NSG-SGM3 humanized mice. Indeed, our model exhibits a mixed Th2/Th17 response and HDM-specific IgE production relevant to allergic severe asthma. All these features were decreased under Der p 2.1 treatment and associated with an increase in Treg frequency in the lungs, consistent with results in non-humanized mice and with an anti-inflammatory effect of Treg cells induced by peptide therapy.

We particularly focused on dendritic and T cell polarization. Dendritic cells are known to be the bridge between innate and adaptative immunity, especially for the induction of allergen-specific Th2 response in asthma notably through type 2 conventional dendritic cells (cDC2) (40). In contrast, tolerogenic IL-10-secreting dendritic cells (IL-10 DC) are responsible for tolerance induction toward an allergen by inducing allergen-specific Treg cells during the late phase of successful AIT (41). As expected, HDM extract increased Th2 and Th17 cell polarization compared to control *in vitro* without affecting Treg or IL-10-secreting dendritic cells, which could be associated with a defect in regulatory function (42). By contrast, *in vivo* we observe an increase in Treg frequency after treatment; this can be partially explained by the fact that *in vitro* the number of cells is largely reduced compared with *in vivo*. Moreover, *in vivo* other elements such as other DC cell or other cytokines may support Treg expansion and survival, which is not present *in vitro*. Our results demonstrate a direct capacity of Der p 2.1 to induce an anti-inflammatory phenotype of DCs (43) in both mouse and humanized mice. This direct effect independent of the presence of allergens may be linked to a potential capacity of Der p 2.1 to exert an anti-inflammatory bystander effect that could prevent inflammation from other allergens. In fact, it is likely that Der p 2.1 is able to drive cell polarization toward tolerance not only to HDM allergen but also to other aggressive agents.

The use of whole allergen could present the inconvenience of being recognized by specific IgE and then induce asthma rather than treating it. Several studies have already shown the clinical efficacy and safety of allergen-modified extract (allergoids) in asthma (44–47). Both allergoids and allergen-derived peptides induce Treg and IL-10-secreting DCs to reestablish a tolerance defect (48–50). However, whereas polymerized allergens called allergoids have lower IgE-binding capacity and Th2 activation while retaining the capacity to induce T cell activation (47, 51), Der p 2.1 can also modulate the Th17 axis, which predominates in the severe form of asthma. Despite their efficacy in decreasing asthma features, allergen-derived peptides may also be improved. Indeed, Martínez and colleagues showed that the combination of several Der p allergen-derived peptides exhibits anti-inflammatory properties (52). Moreover, the use of nanoparticles as adjuvants (53) in allergen-derived peptide immunotherapy could potentially improve their ability to polarize innate and adaptive cells into a tolerogenic phenotype.

We demonstrated that Der p 2.1 immunotherapy abrogates HDM severe allergic asthma features through the induction of tolerogenic DCs in both mice and humanized mice. Despite exhibiting a mixed Th2/Th17 inflammation like severe asthmatic patients, our study remains limited by the specie features. Moreover, Der p 2.1-related DC mechanisms were mainly shown *in vitro* and required *in vivo* studies to confirm these results. Nevertheless, our preliminary results concerning Der p 2.1 effect on the Th17 axis are promising and could be useful for severe asthmatic patients and need further investigations. Thus, allergen-derived immunotherapy could be considered as a promising therapeutic approach to treat allergic asthma, including severe steroid-insensitive forms.

## Data Availability Statement

The original contributions presented in the study are included in the article/**Supplementary Material**. Further inquiries can be directed to the corresponding author.

## Ethics Statement

The studies involving human participants were reviewed and approved by the Nantes University Hospital Ethical Committee and the Committee for the Protection of Patients from Biologic Risks. the Nantes University Hospital Ethical Committee and the Committee for the Protection of Patients from Biologic Risks. All subjects provided written informed consent in accordance with the Declaration of Helsinki. Blood samples were collected from patients included in the EXPRESA cohort study (NCT00721097). The patients/participants provided their written informed consent to participate in this study. The animal study was reviewed and approved by the Ethics Committee on Animal Experimentation of the Pays de la Loire (accreditation number: 9456).

## Author Contributions

MK performed the experiments, analyzed the data, and wrote the manuscript. M-AC provided support for the experiments with mice. CB participated in the analysis of cell populations, and LC gave scientific inputs. AM, VS, and GB designed and elaborated the study and supervised manuscript writing. All authors contributed to the article and approved the submitted version.

## Funding

This work was supported by a grant from the Fondation du Souffle and the Fond de Dotation de Recherche en Santé Respiratoire. This work was supported by the national agency and future investment under the program ANR-16-IDEX-0007 and a grant from “Région Pays de la Loire”.

## Conflict of Interest

The authors declare that the research was conducted in the absence of any commercial or financial relationships that could be construed as a potential conflict of interest.

## Supplementary Material

Supplementary Figure 1 Gating strategy for T cells in humanized mice. Gating strategy to obtain T cells was as follows: blood cells (side scatter *[SSC]-A X forward scatter [FSC]-A)*, single cells *(forward scatter [FSC]-H X [FSC]-A)*, human CD45^+^ cells (anti-human CD45, Biolegend), human CD3^+^ T cells and human CD3^+^ CD4^+^ T cells (anti-human CD3 and anti-human CD4, BD Pharmingen).

Supplementary Figure 2 Demographic and clinical data. *Definition of abbreviations*: FEV1, Forced Expiratory Volume in the first second; ACQ, Asthma Control Questionnaire; NA, non-applicable; BMI, Body Mass Index; OCS, oral corticosteroid; LABA, Long-Acting Beta-Agonist.

Supplementary Figure 3 Irrelevant allergen Bet v 1 and non-specific PMA/Ionomycin stimulation do not activate Th2 and Th17 secreting cell in human PBMCs. **(A–D)** IL-5-secreting (Th2) and IL-17-secreting (Th17) T cell number from PBMCs under Bet v 1 or PMA/Ionomycin re-stimulation. 4 HV and 4 ASTHMA patients per group (**Figures 2E, A–D**). Data are shown as mean ± SEM. **P* <.05, ***P* <.01.

Supplementary Figure 4 Der p 2.1 reduces bronchial remodeling and lung innate inflammation. **(A)** Bronchial epithelium thickness. **(B, C, D)** Epithelium-derived cytokine concentration. six to eight (**Figures E1, A**) and four to six (**Figures E1, B–D**) mice per groups. Data are shown as mean ± SEM. **P* <.05, ***P* <.01, ****P* <.001, *****P* <.0001.
