# Supplementary figures and images for: Der p 2.1 Peptide Abrogates House Dust Mites-Induced Asthma Features in Mice and Humanized Mice by Inhibiting DC-Mediated T Cell Polarization
Source: Front Immunol. 2020 Nov 18;11:565431. doi: 10.3389/fimmu.2020.565431 (PMC7708318; doi:10.3389/fimmu.2020.565431)

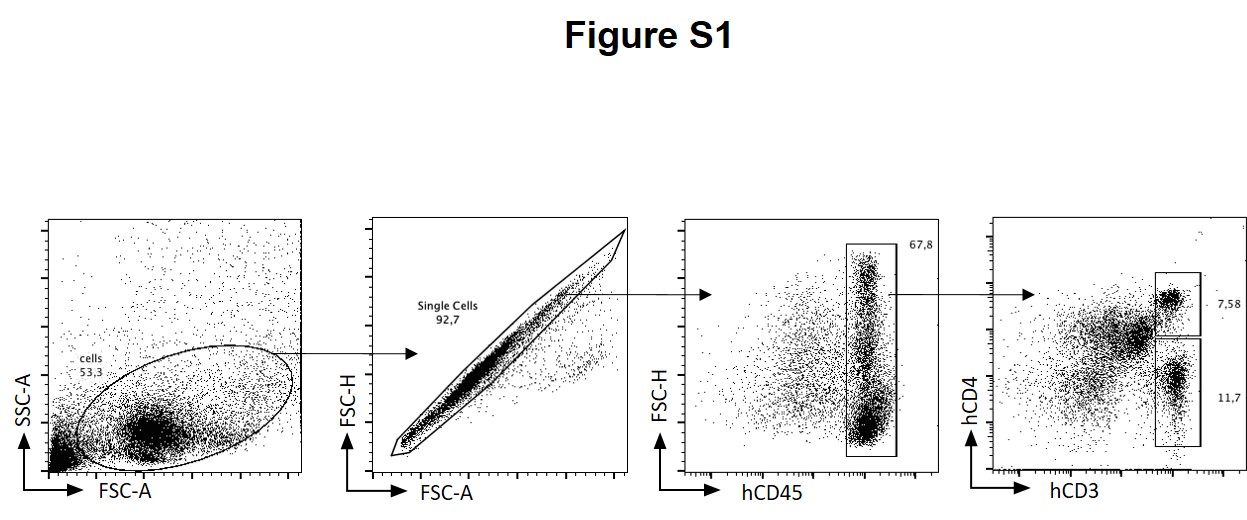

Supplement: Supplementary Figure 1 — Gating strategy for T cells in humanized mice. Gating strategy to obtain T cells was as follows: blood cells (side scatter [SSC]-A X forward scatter [FSC]-A), single cells (forward scatter [FSC]-H X [FSC]-A), human CD45+ cells (anti-human CD45, Biolegend), human CD3+ T cells and human CD3+ CD4+ T cells (anti-human CD3 and anti-human CD4, BD Pharmingen). [file DataSheet_2.zip › Supplementary Figure 1.jpg]

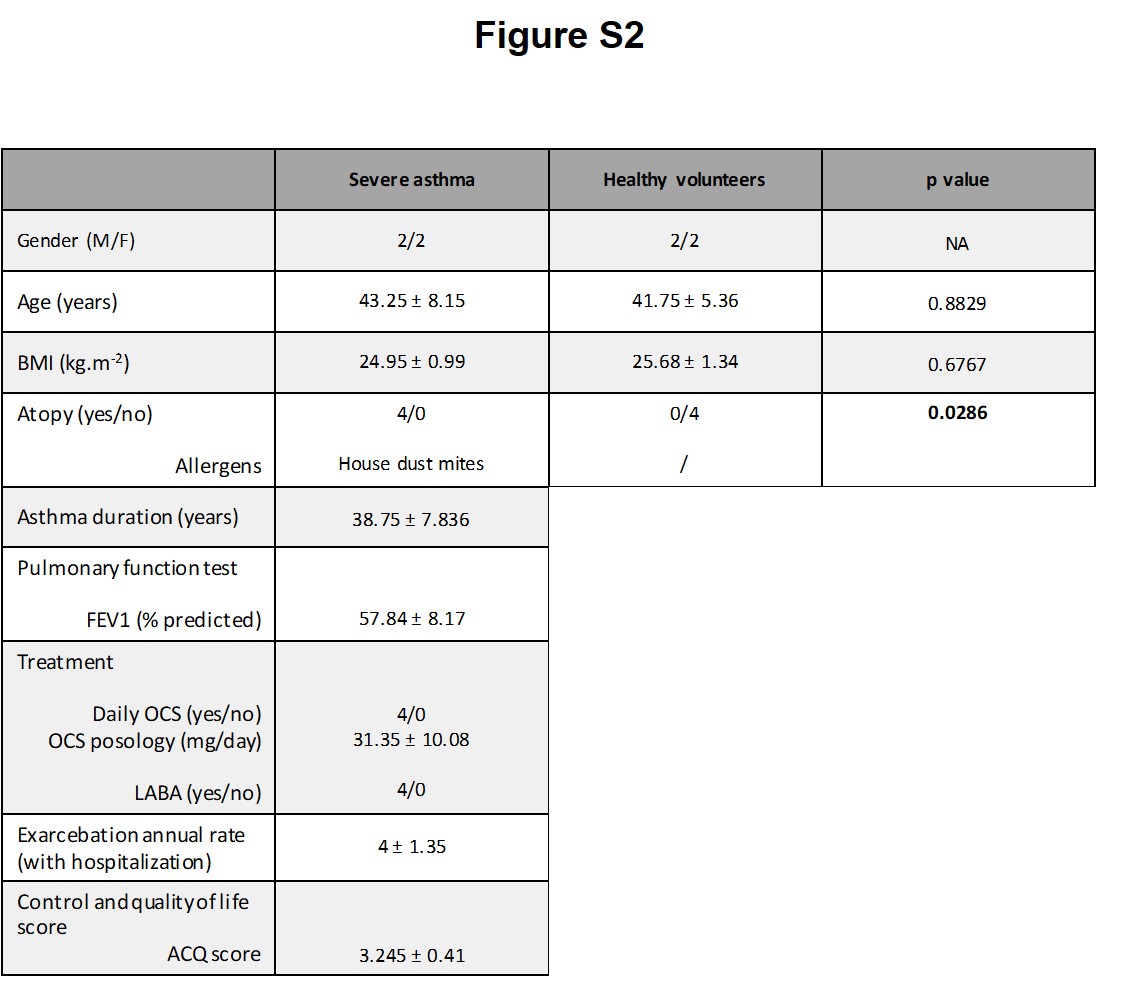

Supplement: Supplementary Figure 1 — Gating strategy for T cells in humanized mice. Gating strategy to obtain T cells was as follows: blood cells (side scatter [SSC]-A X forward scatter [FSC]-A), single cells (forward scatter [FSC]-H X [FSC]-A), human CD45+ cells (anti-human CD45, Biolegend), human CD3+ T cells and human CD3+ CD4+ T cells (anti-human CD3 and anti-human CD4, BD Pharmingen). [file DataSheet_2.zip › Supplementary Figure 2.jpg]

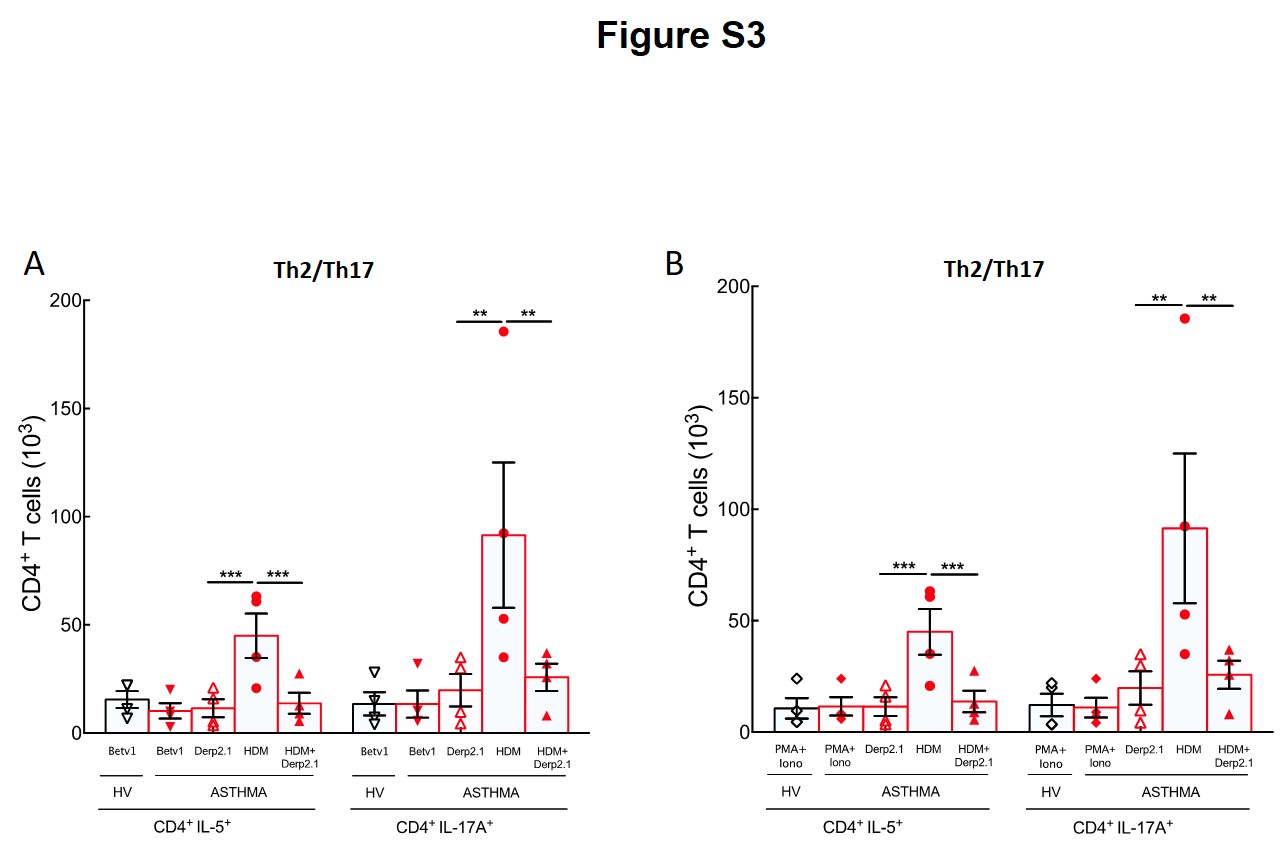

Supplement: Supplementary Figure 1 — Gating strategy for T cells in humanized mice. Gating strategy to obtain T cells was as follows: blood cells (side scatter [SSC]-A X forward scatter [FSC]-A), single cells (forward scatter [FSC]-H X [FSC]-A), human CD45+ cells (anti-human CD45, Biolegend), human CD3+ T cells and human CD3+ CD4+ T cells (anti-human CD3 and anti-human CD4, BD Pharmingen). [file DataSheet_2.zip › Supplementary Figure 3.jpg]

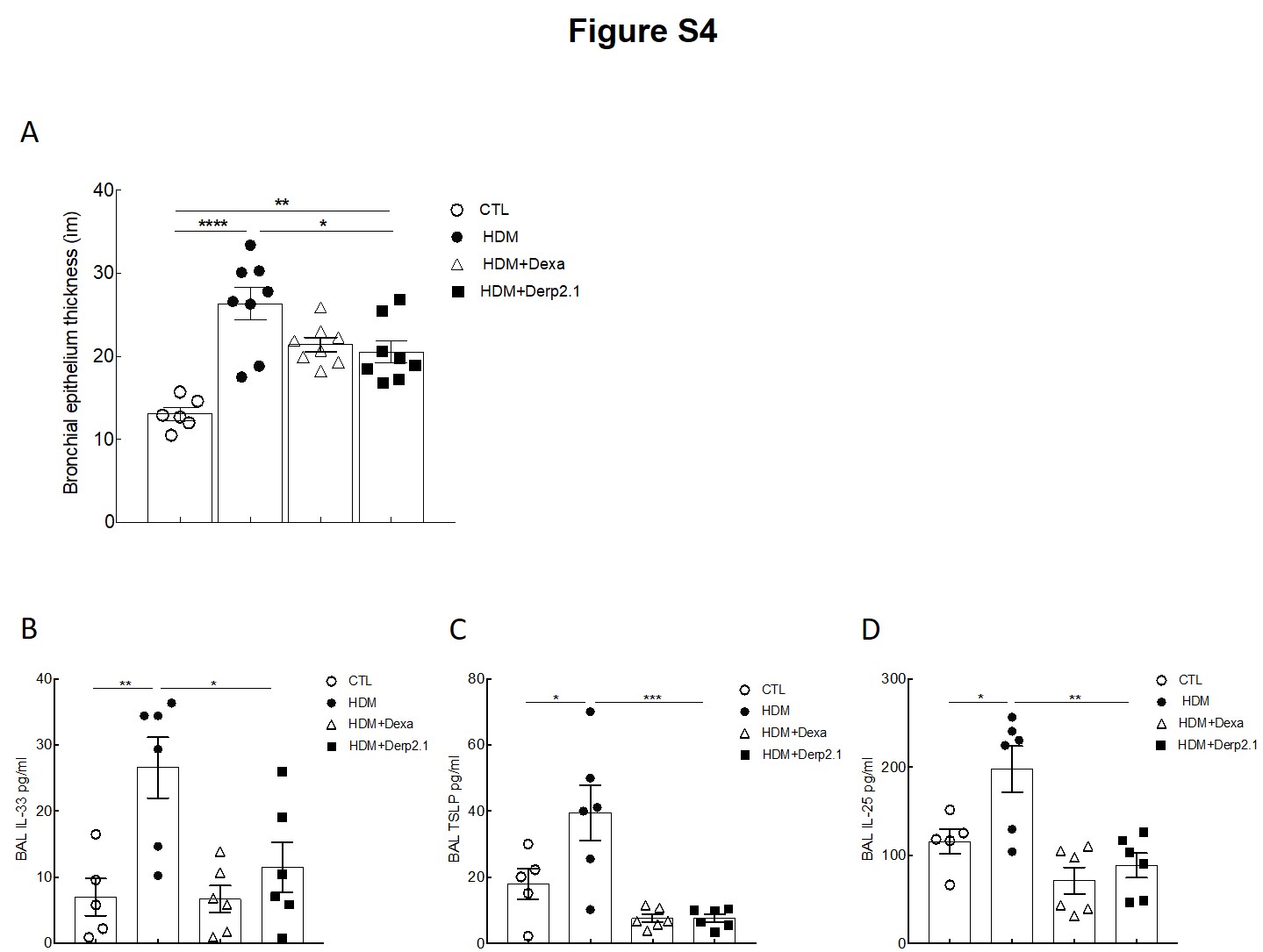

Supplement: Supplementary Figure 1 — Gating strategy for T cells in humanized mice. Gating strategy to obtain T cells was as follows: blood cells (side scatter [SSC]-A X forward scatter [FSC]-A), single cells (forward scatter [FSC]-H X [FSC]-A), human CD45+ cells (anti-human CD45, Biolegend), human CD3+ T cells and human CD3+ CD4+ T cells (anti-human CD3 and anti-human CD4, BD Pharmingen). [file DataSheet_2.zip › Supplementary Figure 4.jpg]
