# Supplementary material for: Der p 2.1 Peptide Abrogates House Dust Mites-Induced Asthma Features in Mice and Humanized Mice by Inhibiting DC-Mediated T Cell Polarization
Source: Front Immunol. 2020 Nov 18;11:565431. doi: 10.3389/fimmu.2020.565431 (PMC7708318; doi:10.3389/fimmu.2020.565431)
Supplement: Supplementary file 2 [file DataSheet_1.docx]

**Supplementary method.**

CD45 (30-F11, BioLegend, Paris, France), F4/80 (BM8, BioLegend), CD11c (Bu15, BioLegend), CD11c (N418, BioLegend), CD3 (17A2, BioLegend), CD3 (HIT3a, BioLegend) , CD4 (GK1.5, BioLegend), CD4 (OKT4, BioLegend), CCR3 (J073E5, BioLegend), Ly6G (1A8, BioLegend), I-A/I-E (M5/114.15.2, BioLegend), HLA-DR (L243, BioLegend), CD103 (2E7, BioLegend), CD25 (PC61, BioLegend), CD25 (BC96, BioLegend), ST2 (DIH4, BioLegend), and CD127 (A019D5, BioLegend), and in the presence of CD16/32 (93, BioLegend), CD16 (3G8, BioLegend) and CD32 (FUN-2, BioLegend) monoclonal antibodies at 1:100. Cells were stimulated for 5 hours with 100 μg/mL of crude HDM preparation and with brefeldin A (Golgi plug, BD Biosciences, Paris, France) at 1:1000. Cells were fixed and permeabilized using a Cytofix/Cytoperm Kit (BD Biosciences, Paris, France) and stained with IL-13 (eBio13A, eBioscience), Gata3 (1A12-1D9, eBioscience), Foxp3 (MF-14, BioLegend), Foxp3 (259D, BioLegend), IL-17A (BL168, BioLegend), IL-17A (TC11-18H10.1, BioLegend), IL-10 (JES5-16E3, BioLegend), IL-10 (JES3-19F1, BioLegend), IL-5 (TRFK5, BioLegend), and RORγt (Q21-559, BD Biosciences), RORγt (Q31-378, BD Biosciences) antibodies at 1:50. The cells were analyzed on a Fortessa X20 cytometer (BD Biosciences, Paris, France).

**Supplementary Figure 1:** Gating strategy for T cells in humanized mice. Gating strategy to obtain T cells was as follows: blood cells (side scatter [SSC]-A X forward scatter [FSC]-A), single cells (forward scatter [FSC]-H X [FSC]-A), human CD45+ cells (anti-human CD45, Biolegend), human CD3+ T cells and human CD3+ CD4+ T cells (anti-human CD3 and anti-human CD4, BD Pharmingen).

**Supplementary Figure 2:** Demographic and clinical data. Definition of abbreviations: FEV1, Forced Expiratory Volume in the first second; ACQ, Asthma Control Questionnaire; NA, non-applicable; BMI, Body Mass Index; OCS, oral corticosteroid; LABA, Long-Acting Beta-Agonist.

**Supplementary Figure 3:** Irrelevant allergen Bet v 1 and non-specific PMA/Ionomycin stimulation do not activate Th2 and Th17 secreting cell in human PBMCs. **(A–D)** IL-5-secreting (Th2) and IL-17-secreting (Th17) T cell number from PBMCs under Bet v 1 or PMA/Ionomycin re-stimulation. 4 HV and 4 ASTHMA patients per group **(Figure 2E, A–D)**. Data are shown as mean ± SEM. *P <.05, **P<.01.

**Supplementary Figure 4:** Der p 2.1 reduces bronchial remodeling and lung innate inflammation. **(A)** Bronchial epithelium thickness. **(B, C, D)** Epithelium derived cytokine concentration. Six to eight **(Figure S1, A)** and four to six **(Figures S1, B–D)** mice per groups. Data are shown as mean ± SEM. *P <.05, **P <.01, ***P<.001, ****P <.0001.
